# Reliability and Validity of the Health-Promoting Lifestyle Profile II Spanish Version in University Students

**DOI:** 10.3390/healthcare12131330

**Published:** 2024-07-03

**Authors:** Rosa Nury Zambrano Bermeo, Catalina Estrada Gonzalez, Eugenia del Pilar Herrera Guerra, Cesar Ivan Aviles Gonzalez

**Affiliations:** 1 School of Health, Universidad Santiago de Cali, Cali 760035, Colombia; rosa.zambrano00@usc.edu.co; 2 School of Health Sciences, Universidad Libre, Sectional Cali, Cali 760035, Colombia; catalina.estradag@unilibre.edu.co; 3 School of Health Sciences, Universidad de Córdoba, Monteria 230002, Colombia; edherrera@correo.unicordoba.edu.co; 4 Department of Nursing, Universidad Popular del Cesar, Valledupar 200002, Colombia; 5 Department of Medical Sciences and Public Health, Università Degli Studi di Cagliari, 09042 Cagliari, Italy

**Keywords:** psychometrics, healthy lifestyle, health promotion, health care students, student health, nursing methodology research, surveys and questionnaires

## Abstract

Aim: The purpose of this study was to assess the reliability and validity of the Spanish version of the *Health Promoting Lifestyle Profile II* (HPLP-II) scale in Colombian university students. Methods: This was a methodological study to verify reliability and construct validity. A total of 763 undergraduate university students in Cali, Colombia, agreed to participate in the study by filling out a form that included information on sociodemographic characteristics and the HPLP-II scale Spanish version. Data were collected between February and June 2021. To determine construct validity, a confirmatory factor analysis was performed, and internal consistency was determined through Cronbach’s alpha. Results: The confirmatory factor analysis of the proposed theoretical model showed that the goodness-of-fit indices of the scale demonstrated an acceptable level of validity nearing an excellent level of fit (χ^2^ = 7168.98; gl = 1268; *p* < 0.001; root mean square error of approximation = 0.08; normed fit index, adjusted goodness-of-fit index = 0.95). Cronbach’s alpha coefficient of the scale was 0.94, and the subscales ranged from 0.68 to 0.89. Conclusions: The HPLP-II Spanish version is a valid and reliable instrument to assess the health-promoting lifestyle profile of university students.

## 1. Introduction 

The World Health Organization (WHO) defines lifestyle as a general way of living based on the interaction between living conditions and individual patterns of behavior [1]. A healthy lifestyle is related to a decrease in chronic non-communicable diseases [2,3]. However, this can be affected by personal factors when confronted with change imposed by environmental needs.

Health promotion is a fundamental approach to improving the wellbeing of individuals and reducing the health risks associated with non-communicable diseases (NCDs). NCDs such as heart disease, diabetes, stroke and cancer are more likely to occur with lifestyle behaviors such as unhealthy diet, tobacco use, alcohol consumption and physical inactivity. Therefore, most NCDs can be prevented simply and affordably by promoting a healthy lifestyle, influenced by promoting health, which involves holding people accountable for self-care [4].

For the purposes of this article, the focus of the present research is on the healthy lifestyles of university students. In this regard, universities have a preponderant role in leading the promotion of healthy lifestyles in students as a part of a formative commitment by implementing promotion and prevention measures to help improve health-promoting behaviors [5]. This is performed to ensure that these students consequently become good health promoters and that these aspects are reflected in the population. However, studies such as Castro et al. [6] pointed out that the university does not exercise the role of promoting healthy lifestyles and the abandonment of harmful styles for university students’ health.

University students have unhealthy lifestyles due to eating habits that do not conform to established recommendations, and they are at risk of developing atypical eating disorders [7]. The college experience is often hectic and demanding, affecting young people’s lifestyles in unhealthy ways. Some of the most common reasons include packed schedules of classes, homework, exams and extracurricular activities. In addition, the convenience of unhealthy fast food options, long study hours, academic pressure, responsibilities and expectations can generate high levels of stress. On the other hand, social life in college often involves parties and events where alcohol and tobacco are consumed [8]. And Belmonte Cortes et al. [9] reported that university students do not engage in physical activity or any type of sport in their free time.

To fully understand the lifestyle of university students and elucidate the importance of the periodic evaluation of the lifestyle profile in this population group and the main purpose of this research, it is necessary to first address the central aspects of the Health Promotion Model (HPM).

Nola Pender, through the HPM, provides a conceptual framework for studying health-promoting behaviors and the way in which these might relate in order to improve health and wellbeing. Furthermore, it demonstrates that the external environment influences the increase or decrease in engagement or participation in health-promoting behaviors. It enables the examination of variables influencing the individual, thereby relating the effect to the activity, the commitment to an action plan, and the immediate opposing demands and preferences. It also contributes to the empirical demonstration of health-promoting behaviors and facilitates the generation of demonstrable hypotheses [10]. 

According to the HPM, healthy behavior is determined by the following individual characteristics and experiences that affect health actions: (1) personal factors categorized as biological, psychological and psychosocial; (2) socio-cognitive variables such as perceived benefits and barriers to action, perceived self-efficacy, emotional and affective elements; and (3) interpersonal and contextual factors, such as perceived influences during the socialization process, social norm, the relationship to the contexts in which the individual finds themselves [10]. 

The theoretical assumptions of the HPM emphasize the active role in managing health-promoting behaviors, and the theoretical statements illustrate the multifaceted nature of the individual interacting with the environment when attempting to achieve a state of health. The model is oriented toward motivating the desire to enhance wellbeing and actualize human potential so that the individual engages in health-promoting behaviors. Health-promoting behaviors are the result of action directed toward positive health outcomes such as optimal wellbeing, personal fulfillment and productive living. Examples of health-promoting behaviors include maintaining a healthy diet, engaging in regular physical exercise, managing stress, getting adequate rest, spiritual growth and building positive relationships [10]. 

Based on the HPM, Walker et al. [11] developed an instrument called a health-promoting lifestyle profile to study this behavior in healthy and unwell adults. Psychometric tests of Nola Pender’s instrument (HPLP-II) in the English and Spanish versions demonstrate that it is a valid and reliable instrument for research within the framework of the HPM. The results of studies conducted with the HPM and the application of the HPLP-II scale, according to Heydari and Khorashadizadeh [12] and Pender [13], have demonstrated the predictive ability of the model for lifestyle health promotion. Since its publication, it has been widely used in several countries, and it has been translated and validated in several languages and different population groups, verifying its psychometric properties [14,15,16,17]. 

This study used the Spanish version of Walker et al. [18] called Lifestyle Questionnaire II. The HPLP-II questionnaire was developed with the theoretical framework of the HPM [13]. It is considered valid and reliable for use in research to measure the health-promoting lifestyle construct [19]. It consists of 52 items and six subscales as follows: responsibility for health (nine items: 3, 9, 15, 21, 27, 33, 39, 45, 51), physical activity (eight items: 4, 10, 16, 22, 28, 34, 40, 46, 51), nutrition (nine items: 2, 8, 14, 20, 26, 3, 38, 44, 50), spiritual growth (nine items: 6, 12, 18, 24, 30, 36, 42, 48, 52), interpersonal relationships (nine items: 1, 7, 13, 19, 25, 31, 37, 43, 49), and stress management (eight items: 5, 11, 17, 23, 29, 35, 41, 47). The statements are scored on a four-point Likert scale ranging from never (1 point) to routinely (4 points).

Likewise, several studies have used the HPLP-II scale to assess health-promoting behaviors and lifestyle profile factors in university students in the health care field, such as medicine and nursing [20,21,22,23,24]. When analyzing the six components of the health-promoting lifestyle profile and their association with sociodemographic and health factors, differences have been identified in terms of sex—with regard to interpersonal relationships in particular—and physical activity, economic level, year of study, and family structure, among others. Studies showed that most of the students had unhealthy eating habits and low levels of physical activity.

University programs in health faculties are favorable scenarios for teaching health-promoting behaviors to students, who are expected to be responsible for promoting healthy lifestyles in the general population to prevent NCDs. Interestingly, studies reveal that students have unhealthy lifestyles despite being taught about these issues. The findings demonstrate the importance of conducting periodic assessments of the lifestyle profile of university students.

Few studies in Colombia have explored the health-promoting lifestyle profile among university students. The main limitation may be that the HPLP-II scale is not currently validated. Therefore, the aim of the present investigation was to determine the validity and reliability of the HPLP-II scale in Colombian university health students.

## 2. Materials and Methods 

A cross-sectional study included the evaluation of the construct validity and internal consistency of the Spanish version of the HPLP-II scale. First, the linguistic equivalence of the scale in the Colombian context was verified. Second, data were collected using the original Spanish version scale and analyzed to confirm validity and reliability.

### 2.1. Sample

The participants of this study were 763 Colombian students at four universities in Valle del Cauca between February and June 2021. The sample was selected by convenience considering the following inclusion criteria: undergraduate students from the health sciences faculty of four universities in Valle del Cauca. Students enrolled in other faculties offered by the university and postgraduate students were excluded.

### 2.2. Data Collection

Data were collected online between February and June 2021. The researchers distributed the data collection instruments to the participants via email and asked them to complete the information, with prior informed consent, anonymously and individually. Participants did not receive remuneration for participation in the study.

### 2.3. Data Collection Instruments 

#### 2.3.1. Information Form Including Sociodemographic Characteristics

This form was developed by the researchers. It includes sociodemographic data of the participants, such as age, sex, ethnicity, socioeconomic status and academic program.

#### 2.3.2. Health-Promoting Lifestyle Profile-II

The HPLP-II scale was developed by Walker et al. [11]. It consists of 52 items and six subscales. The overall score was obtained by calculating the mean of the responses to the items. Subscale scores are obtained by calculating the mean of the responses to the items in each of the subscales [25].

The original version of the HPLP-II scale has content validity established through a literature review along with expert validity, construct validity by exploratory factor analysis that confirmed a six-dimensional model, convergent validity (r = 0.678), criterion validity with significant correlations with concurrent measures of perceived health status, and quality of life (rs = 0.269–0.491). Reliability testing proved internal consistency for the total scale with a Cronbach’s alpha of 0.94, and for the subscales, Cronbach’s alpha ranged from 0.79 to 0.87 [25].

The HPLP-II scale Spanish version is also considered a valid and reliable instrument [26,27]. It has been used in Colombia to conduct studies on health-promoting behaviors in postmenopausal women [28], in women within social programs [29], in university students [30,31], and in patients with chronic obstructive pulmonary disease [32]. In the literature review, no studies were found on the construct validity of the Colombian Spanish version of the Lifestyle Questionnaire II. Therefore, the abovementioned points highlight the importance of this study.

### 2.4. Linguistic Equivalence of the Scale

The linguistic equivalence of the HPLP-II scale in Spanish (Spain) with Colombian Spanish was verified by means of the cross-cultural adaptation method, with the support of a linguist, in order to guarantee compliance with the semantic and linguistic criteria. The scale was applied to a group of students (n = 20) and university professors (n = 15), and the comprehension of the items was checked, and no problematic items were found. Therefore, it was not necessary to make changes to the original HPLP-II scale.

### 2.5. Data Analysis

The data were processed using SPSS 22 and AMOS 22 statistical software. Descriptive statistical analysis of the sociodemographic variables (mean, standard deviation and frequencies) was performed. The adequacy of the sample size for the factor analysis was verified by applying the Kaiser–Meyer–Olkin test (KMO > 0.7) and Bartlett’s test of sphericity (*p* value < 0.05). Confirmatory factor analysis (CFA) was performed with the weighted least squares mean and variance adjusted method (WLSMV). The chi-square goodness of fit test, adjusted goodness of fit index (AGFI), normed fit index (NFI) and non-normalized fit index (NNFI) were evaluated. A value of 0.90 indicates an acceptable fit, and 0.95 indicates an excellent fit. For the root mean square error of approximation (RMSEA) and standardized root mean square error (SRMR), a value of 0.08 confirms an acceptable fit, and 0.05 indicates an excellent fit [33]. Cronbach’s alpha coefficient (>0.7) was used to determine internal consistency [34].

### 2.6. Ethical Considerations

They were given compliance with international ethical guidelines [35] and ethical norms for research in Colombia [36]. The study obtained written permission from the author of the HPLP-II scale. Permission was also obtained from the ethics committee of the Universidad Santiago de Cali (Date: 26 June 2020), minute no. 01. All participants gave their consent, were assured of their voluntary participation, and were made aware of the objectives of the study and what was expected of them.

## 3. Results

### 3.1. Characteristics of Participants 

Table 1 shows the characteristics of the study participants. The mean age of the undergraduate student participants in the study was 21.7 ± 4.2 years (age range: 18–36 years); 78.9% were women. In addition, the majority were of mixed race (56.1%), and 35.5% were from socioeconomic strata three. Students from different academic programs participated, mainly from nursing (38.8%) and medicine (29%).

### 3.2. Construct Validity Test 

#### 3.2.1. Reliability Test

Table 2 shows that the item–total correlations in all cases are greater than 0.20; furthermore, if any particular item is eliminated, this does not cause an increase in Cronbach’s alpha, so it is not necessary to eliminate any of them. Accepting the reliability of the instrument with the 52 items.

On the other hand, Table 3 shows the correlation results of the subscales, which indicate statistically significant correlations between all the subscales. Regarding the reliability of the scale, an excellent Cronbach’s alpha coefficient of 0.94 was obtained. For the dimensions studied, good values were obtained for physical activity (0.89), spiritual growth (0.87) and social responsibility (0.84); acceptable for interpersonal relationships (0.79) and stress management (0.76); and acceptable but questionable for nutrition (0.68).

#### 3.2.2. Confirmatory Factor Analysis (CFA)

The results of the obtained Kaiser–Meyer–Olkin test (KMO = 0.95) and Bartlett’s sphericity test (*p* < 0.001) indicated the adequacy of sampling in which the criteria for performing a factor analysis were met.

**Figure 1:**
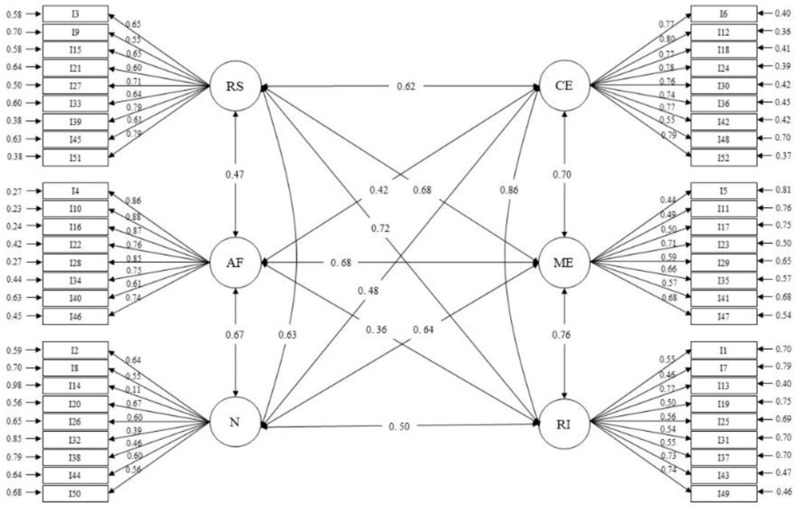
Structural diagram of the confirmed model and factor loading. Note: HR = health responsibility; PA = physical activity; N = nutrition; SG = spiritual growth; IR = interpersonal relationships; SM = stress management.

The structure of the original HPLP-II scale was tested for confirmation in the Colombian university student sample. Table 2 provides the fit CFA indices of the study for the six-factor model of the Spanish version. The analysis of the proposed theoretical model showed that all factor loadings were significant (*p* < 0.05). A significant scale index was obtained (χ^2^ = 7168.98; gl = 1268; *p* < 0.001); however, the value of CMIN/DF = 5.60, resulting from dividing χ^2^ = 7168.98 by gl = 1268, is close to the desirable value (≤5). Likewise, the model evaluated in the CFA shows consistency in the other metrics. The absolute goodness-of-fit index values of the scale demonstrated an acceptable level of validity (RMSEA = 0.078; SRMR = 0.079), as did the incremental fit indices (NFI = 0.956; NNFI = 0.954; AGFI = 0.95). This demonstrates the existence of significant evidence to infer that the instrument is adequate. Data on the confirmed model and factor loadings of the subscales are presented in Table 2 and Figure 1.

The CFA of the HPLP-II scale in this study reported a theoretical model in which the 52 items and the six subscales defined in the original model were retained. Each item saturates only on the factor dimension of which it is assumed to be a valid indicator. Each variable saturates only on the common factor that measures the correlated common factors. More than three items per factor are available, which substantially improves the precision of the estimates. This allows us to highlight some of the most relevant aspects of model specification and identification.

In the CFA, it is necessary to observe the factor loadings that allow for the correlation between variables and factors to be established [37]. The closer these variables are to one, the greater the Raubenheimer correlation [38]. For the particular case of this research, the CFA is as shown in Figure 1, where at the end are six variables with which the relationship between the constructs of the model could be established.

The results in Table 3 show a higher correlation between the subscales interpersonal relationships and spiritual growth (r = 0.86), stress management and spiritual growth (r = 0.76), interpersonal relationships and health responsibility (r = 0.72), stress management and interpersonal relationships (r = 0.70), and physical activity and stress management (0.68). The stress management scale was highly correlated with all subscales (r > 0.64). These correlations are similar to the findings of other studies [39].

Because the model presented has latent or unobserved variables, it is necessary to identify each of them with a statistical value to calculate estimates of their effects. The estimated values evaluate a parameter that characterizes the population through a sample [40]. The goodness-of-fit criteria of the model in this study were evaluated from two perspectives: absolute fit and incremental fit. The reported absolute goodness of fit was significant (χ^2^ = 7168.98; gl = 1268; *p* < 0.001; RMSEA = 0.08), thus establishing that the relationships between the constructs and hypotheses have significance. The incremental model fit measures allowed for comparing the proposed model with the existing model; the AGFI and NFI were >0.09 on the global scale and on the six dimensions. The AGFI with values close to ≥0.90 shows a better fit of the Martinez model [41]. The NFI is considered an acceptable value if it is >0.90.

### 3.3. Reliability Test

Table 3 presents the correlation results of the subscales, indicating statistically significant correlations between all subscales. Cronbach’s alpha coefficient of the scale was 0.94, and the subscales ranged from 0.68 to 0.89.

## 4. Discussion 

The purpose of this study was to evaluate the reliability and construct validity of the Spanish version of the HPLP-II scale in Colombian university students. In the factor analysis, the six subscales of the original version of the proposed theoretical model were ratified. The scale’s goodness-of-fit indices showed an acceptable level of validity and a nearly excellent level of fit. The Cronbach’s alpha coefficient of the scale was 0.94, which indicates good internal consistency. These findings are consistent with other studies conducted in different countries and languages, demonstrating that the HPLP-II scale with six subscales is a valid and reliable instrument to assess the health-promoting lifestyle profile of university students [42,43,44,45].

In the study by Walker et al., the reliability coefficient alpha for the total scale of the HPLP-II (original version) was 0.93. The results of construct validity (CFA) were not reported. In the six-dimensional model with the 52-item Spanish version tested by Kuster et al. [46], the alpha coefficients were 0.94 for the total scale and ranged between 0.64 and 0.89 for the subscales, values similar to those obtained in this study, indicating reliability. This model was also tested in the study by Enriquez et al. [47], with a sample of Mexican university students, where it presented an acceptable fit that explains 49.93% of the variance. The RMSEA value was 0.08, which is equal to that found in this study, indicating a moderate fit in the two models evaluated, so it can be considered a culturally valid instrument for evaluating healthy lifestyles in young university students.

According to Messick [48], construct validity is the main type of validity. It is the unifying concept that integrates content and criterion validity considerations into a common framework for testing hypotheses about theoretically relevant relationships. The health-promoting lifestyle construct measured by the HPLP-II is defined as a multidimensional pattern of self-initiated actions and perceptions that serve to maintain or enhance an individual’s level of wellbeing and self-fulfillment [49,50]. 

The results of the study suggest the benefits of multicomponent educational interventions to improve health-promoting behaviors in university students, based on Pender’s HPM, developed from the analysis of the six components of the health-promoting lifestyle profile that make up the HPLP-II scale: responsibility for health, physical activity, nutrition, spiritual growth, interpersonal relationships and stress management. In this sense, Lee et al. [51] consider that health-promoting behaviors should be an integral part of the lifestyle of university students, who should adopt healthy habits during youth since it is difficult to change unhealthy habits in adulthood. However, Bryer et al. [52] recommend that universities provide scenarios that promote the lifestyle profile and health of young university students, developing actions that contribute to health promotion in the curricula to ensure that they acquire the knowledge and skills to maintain health and achieve behavioral changes.

While psychometric studies prove that the health-promoting lifestyle profile exhibits a multidimensional pattern, interventions to improve lifestyle in college students have focused primarily on improving nutrition and physical activity [53,54,55], and few studies have intervened in stress management [56]. Knowledge gaps indicate that more research is needed to evaluate the effectiveness of interventions that integrate the six dimensions of the HPLP-II. It is hoped that the HPLP-II can be used as a psychometric tool to measure the effectiveness of multidimensional interventions in Colombian university students developed by multidisciplinary teams, with the purpose of improving the health and wellbeing of young people that have a significant impact on the reduction in chronic non-communicable diseases at an early age.

## 5. Study Limitations

This research has some limitations related to methodological studies. This study was conducted in four universities in Valle del Cauca, Colombia, and it did not include university students from other regions of the country. It should be taken into account that cultural differences may affect the psychometric properties of the scale, so it is recommended that the generalization of this study be examined in relation to the different regions of the country.

## 6. Conclusions

Psychometric tests on the six dimensions of the HPLP-II scale, Spanish version, applied to Colombian university students, showed results that support construct validity and reliability. Those interested in assessing the health-promoting lifestyle profile of university students are encouraged to use this scale, and researchers are encouraged to contribute to additional psychometric tests. Further studies in other cultural groups are needed to confirm the evidence suggested in this analysis.

## Figures and Tables

**Table 1:** Characteristics of study participants (n = 763).

Characteristics	N (%)
**Sex**	
Male	161 (21.1)
Female	602 (78.9)
**Age (years)**	
Mean ± SD	21.73± 4.29
**Ethnicity**	
White	181 (23.7)
Indigenous	19 (2.5)
Mestizo	428 (56.1)
Mulatto	17 (2.2)
Black	116 (15.2)
Raizal	2 (0.3)
**Socioeconomic stratum**	
1	90 (11.8)
2	244 (32)
3	271 (35.5)
4	114 (14.9)
5	36 (4.7)
6	8 (1)
**Academic program**	
Pre-hospital care	2 (0.3)
Nursing	296 (38.8)
Physiotherapy	55 (7.2)
Phonoaudiology	18 (2.4)
Surgical instrumentation	138 (18.1)
Dental mechanics	1 (0.1)
Medicine	221 (29)
Dentistry	13 (1.7)
Psychology	16 (2.1)
Pharmacy Regency	1 (0.1)
Respiratory therapy	2 (0.3)

Note: SD = standard deviation.

**Table 2:** Six-factor measurement model of the HPLP-II and its fit indexes.

Scale	Indicators	Mean ± SD	Item–Total Correlation	Corrected Item–Total Correlation	Factorial Loading (*p* Value)	
HR	Item 3	2.41 ± 0.66	0.51	0.49	0.65 (<0.001)	
	Item 9	2.23 ± 0.72	0.46	0.45	0.55 (<0.001)	
	Item 15	2.53 ± 0.62	0.50	0.48	0.65 (<0.001)	
	Item 21	2.25 ± 0.71	0.49	0.48	0.60 (<0.001)	
	Item 27	2.38 ± 0.67	0.54	0.52	0.71 (<0.001)	
	Item 33	2.51 ± 0.67	0.52	0.50	0.63 (<0.001)	
	Item 39	2.37 ± 0.69	0.63	0.61	0.79 (<0.001)	
	Item 45	1.77 ± 0.76	0.47	0.47	0.61 (<0.001)	
	Item 51	2.64 ± 0.55	0.61	0.58	0.79 (<0.001)	
PA	Item 4	2.04 ± 0.79	0.56	0.55	0.86 (<0.001)	
	Item 10	2.14 ± 0.79	0.54	0.52	0.88 (<0.001)	
	Item 16	2.17 ± 0.75	0.60	0.59	0.87 (<0.001)	
	Item 22	2.15 ± 0.78	0.56	0.56	0.76 (<0.001)	
	Item 28	2.22 ± 0.76	0.57	0.56	0.86 (<0.001)	
	Item 34	2.27 ± 0.75	0.56	0.55	0.75 (<0.001)	
	Item 40	1.95 ± 0.80	0.43	0.42	0.61 (<0.001)	
	Item 46	1.98 ± 0.81	0.54	0.53	0.74 (<0.001)	
N	Item 2	2.17 ± 0.69	0.45	0.45	0.65 (<0.001)	
	Item 8	2.22 ± 0.73	0.39	0.38	0.55 (<0.001)	
	Item 14	1.88 ± 0.78	0.10	0.10	0.11 (<0.05)	
	Item 20	2.15 ± 0.68	0.48	0.47	0.67 (<0.001)	
	Item 26	2.27 ± 0.72	0.43	0.42	0.60 (<0.001)	
	Item 32	2.25 ± 0.71	0.31	0.30	0.39 (<0.001)	
	Item 38	2.66 ± 0.54	0.34	0.33	0.45 (<0.001)	
	Item 44	2.03 ± 0.81	0.44	0.43	0.60(<0.001)	
	Item 50	2.60 ± 0.59	0.42	0.40	0.556(<0.001)	
CE	Item 6	2.76 ± 0.50	0.61	0.58	0.77 (<0.001)	
	Item 12	2.88 ± 0.37	0.55	0.51	0.80 (<0.001)	
	Item 18	2.59 ± 0.57	0.54	0.50	0.77 (<0.001)	
	Item 24	2.71 ± 0.53	0.61	0.57	0.78 (<0.001)	
	Item 30	2.85 ± 0.39	0.59	0.56	0.76 (<0.001)	
	Items 36	2.58 ± 0.58	0.61	0.59	0.74 (<0.001)	
	Items 42	2.87 ± 0.36	0.57	0.53	0.77 (<0.001)	
	Items 48	2.37 ± 0.75	0.46	0.45	0.55 (<0.001)	
	Items 52	2.72 ± 0.49	0.65	0.63	0.79 (<0.001)	
IR	Items 1	2.31 ± 0.63	0.43	0.40	0.55 (<0.001)	
	Item 7	2.90 ± 0.36	0.32	0.30	0.47 (<0.001)	
	Items 13	2.83 ± 0.42	0.56	0.53	0.76 (<0.001)	
	Items 19	2.46 ± 0.62	0.41	0.40	0.50 (<0.001)	
	Items 25	2.53 ± 0.66	0.44	0.42	0.56 (<0.001)	
	Items 31	2.61 ± 0.62	0.42	0.40	0.55 (<0.001)	
	Items 37	2.43 ± 0.67	0.45	0.43	0.55 (<0.001)	
	Items 43	2.43 ± 0.69	0.57	0.55	0.73 (<0.001)	
	Items 49	2.81 ± 0.43	0.57	0.55	0.73 (<0.001)	
SM	Items 5	2.42 ± 0.61	0.37	0.36	0.44 (<0.001)	
	Items 11	2.34 ± 0.69	0.43	0.42	0.49 (<0.001)	
	Items 17	2.59 ± 0.57	0.44	0.42	0.50 (<0.001)	
	Items 23	2.58 ± 0.62	0.58	0.56	0.71 (<0.001)	
	Items 29	1.79 ± 0.78	0.49	0.49	0.59 (<0.001)	
	Items 35	2.37 ± 0.66	0.56	0.54	0.66 (<0.001)	
	Items 41	1.69 ± 0.76	0.46	0.45	0.57 (<0.001)	
	Items 47	2.06 ± 0.74	0.57	0.56	0.68 (<0.001)	
**Adjustment indices**						
**Absolute adjustment measures**				**Incremental adjustment measures**		
	**χ^2^; gl (p)**	**RMSEA** **(CI 90%)**	**SRMR**	**NFI**	**NNFI**	**AGFI**
Acceptable level of adjustment	-	≤0.08	≤0.08	>0.90	≥0.90	≥0.90
Measure	7168.98; 1268 (<0.001)	0.078 (0.076; 0.080)	0.079	0.956	0.954	0.95

Note: RS = health responsibility; PA = physical activity; N = nutrition; SG = spiritual growth; IR = interpersonal relationships; SM = stress management; χ^2^ = chi-square; gl = degrees of freedom; RMSEA = root mean square error of approximation; CI = confidence interval; SRMR = standardized root mean square error; NFI = standardized fit index; NNFI = nonstandardized fit index; AGFI = adjusted goodness-of-fit index; SD = standard deviation.

**Table 3:** Correlation of the subscales and Cronbach’s alpha coefficients.

	HR r	PA r	N r	SG r	IR r	SM r
HR	1					
PA	0.47	1				
N	0.63	0.67	1			
SG	0.62	0.42	0.48	1		
IR	0.72	0.36	0.50	0.86	1	
SM	0.68	0.68	0.64	0.76	0.70	1
Cronbach’s alpha	0.84	0.89	0.68	0.87	0.79	0.76
Overall Cronbach’s alpha	0.94					

Note: HR = health responsibility; PA = physical activity; N = nutrition; SG = spiritual growth; IR = interpersonal relationships; SM = stress management; r = Pearson’s correlation coefficient, *p* < 0.01.

## Data Availability

All data generated or analyzed during this study are included in this published article.
